# Spectral Analysis of Naturally Occurring Methylxanthines (Theophylline, Theobromine and Caffeine) Binding with DNA

**DOI:** 10.1371/journal.pone.0050019

**Published:** 2012-12-07

**Authors:** Irudayam Maria Johnson, Halan Prakash, Jeyaguru Prathiba, Raghavachary Raghunathan, Raghunathan Malathi

**Affiliations:** 1 Department of Physiology, University of Tennessee Health Science Center, Memphis, Tennessee, United States of America; 2 National Centre for Ultrafast Processes, Taramani Campus, University of Madras, Chennai, India; 3 Department of Genetics, Taramani Campus, University of Madras, Chennai, India; 4 Department of Organic Chemistry, Guindy Campus, University of Madras, Chennai, India; University of Quebect at Trois-Rivieres, Canada

## Abstract

Nucleic acids exist in a dynamic equilibrium with a number of molecules that constantly interact with them and regulate the cellular activities. The inherent nature of the structure and conformational integrity of these macromolecules can lead to altered biological activity through proper targeting of nucleic acids binding ligands or drug molecules. We studied the interaction of naturally occurring methylxanthines such as theophylline, theobromine and caffeine with DNA, using UV absorption and Fourier transform infrared (FTIR) spectroscopic methods, and especially monitored their binding affinity in the presence of Mg^2+^ and during helix-coil transitions of DNA by temperature (*T_m_*) or *pH* melting profiles. The study indicates that all these molecules effectively bind to DNA in a dose dependent manner. The overall binding constants of DNA-theophylline = 3.5×10^3^ M^−1^, DNA-theobromine = 1.1×10^3^ M^−1^, and DNA-Caffeine = 3.8×10^3^ M^−1^. On the other hand *T_m_/pH* melting profiles showed 24–35% of enhanced binding activity of methylxanthines during helix-coil transitions of DNA rather than to its native double helical structure. The FTIR analysis divulged that theophylline, theobromine and caffeine interact with all the base pairs of DNA (A-T; G-C) and phosphate group through hydrogen bond (H-bond) interaction. In the presence of Mg^2+^, methylxanthines altered the structure of DNA from B to A-family. However, the B-family structure of DNA remained unaltered in DNA-methylxanthines complexes or in the absence of Mg^2+^. The spectral analyses indicated the order of binding affinity as “caffeine≥theophylline>theobromine” to the native double helical DNA, and “theophylline≥theobromine>caffeine to the denatured form of DNA and in the presence of divalent metal ions.

## Introduction

**Figure 1:**
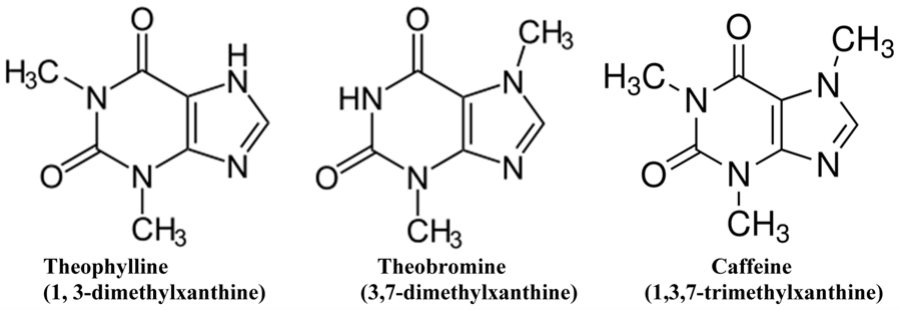
The chemical structure of naturally occurring methylxanthines.

The emphasis on studying the interaction of methylxanthines such as theophylline, theobromine and caffeine (Fig. 1) with nucleic acids is mainly because of a) its dietary consumption b) their use as therapeutic agents. Interestingly these xanthine derivatives have interactions with steroid-receptor complex, DNA, RNA, adenosine receptor, protein kinases, and neurological behavior [1]–[16] which are reckoned to be pivotal for their ability to modulate the biochemical reactions by interacting with the nucleic acids or through cell signaling molecules.

While probing the spectroscopic analysis of methylxanthines interaction with nucleic acids, it has been understood that caffeine known to interact with 5′-adenosine monophosphate and poly riboadenylate by a parallel arrangement outside-stacked self-association to DNA bases [2], [3], and report from Nafisi et.al, indicate that caffeine and theophylline bind to DNA in aqueous solution [17]. However a comparative analysis of caffeine, theophylline with the other structurally related compounds like theobromine has not yet been shown to understand their variance in binding efficacy with DNA, as all of them are having vital cellular activities. Moreover, the current study deals the binding interaction of all these three methylxanthines with DNA in the presence of divalent metal ions and during helix-coil transition state holding some key rationales are explicitly explored in detail.

As far as the importance of theobromine is concerned it has been shown that theobromine enhanced the antitumor activity of adriamycin with reduced toxicity [18], [19]. It has also been reported that caffeine and theobromine inhibited the doxorubicin efflux from tumor cells and increased the tumoricidal activity with reduced side effect [8]. From our earlier reports it could be understood that since xanthine derivatives can interact with DNA, they can reduce the DNA-directed toxicity of certain intercalating dyes such as ethidium bromide, acridine orange and antitumor agents like cisplatin, novantrone, actinomycin D etc [16]. Moreover, co-administration of methylxanthines in cancer therapy used for the enhancement of anti-tumor agent's activity and serving as candidates for radiosensitization are promising baseline for developing methylxanthines as potential secondary enhancers for future clinical trial [20]–[23]. It is worthwhile to mention here that caffeine and theophylline decrease the replication of the virus HIV-1 strain [24]. We have also demonstrated that methylxanthines can modulate the self-splicing activity of group I intron, showing both theophylline and theobromine relatively reduce the splicing activity of group I intron as compared to that of the control self-splicing reaction. However, caffeine, with structural difference of a single methyl group at the N-7 position, was not effective, and thus forming the baseline for the development of splicing inhibitors with possible role of RNA as drug target [25] (Fig. 1).

The rationales for studying the interaction of methylxanthines in the presence of divalent metal ions are mainly due to a fact that these divalent metal ions are being preferred for many enzymatic activities and also needed for many small molecule drugs and antibiotics for their effective biding to DNA or RNA or cellular proteins. The recent trends on the binding interaction of metal ions with cellular components by itself or together with other drug molecules bring out either beneficial or non-beneficial cellular effects. For instance higher DNA-acting efficacy is noticed for the DNA-binding anticancer agents such as Chromomycin A3 in the presence of divalent metal ions [26]. Divalent metal ions such as magnesium is the preferred divalent metal ion for efficient and specific cleavage reaction of I-BmoI endonucleases [27].The activity of “Core A” transporter protein depends on the binding of divalent metal ions where the interaction of magnesium ions to its interhelical loops is explored in detail [28]. On the other hand studying the binding interactions and the affinity of some of the non-beneficial divalent metal ions in the cellular system is highly helpful to identify their toxicities to vital cells. In this respect the divalent metal ions such as Pb^2+^ interact with the His-330 and His-362 residues in neurological Tau protein causing the fibril formation might lead to pathophysiological significance of Alzheimer disease [29]. However the metal ionophore treatment alleviates the Alzheimer disease pathology in mouse model [30]. Thus metals and their counter parts are found to modulate the vital cellular events need to be focused for refining the cellular events to be a beneficial interaction.

The validation behind the DNA melting studies are owing to the fact that DNA stabilization occurs through several physico-chemical factors like base stacking, hydrogen bonding, hydrophobic, electrostatic, van der Waals interactions etc., do not provide the accessibility for gene expression. However the DNA energetics effect on its structure allow the gene expression and genome organization [31] to be an accessible denominator for the exploitation of cellular function to be a beneficial event through proper targeting by small molecule drugs triggered the focus for the preferential binding of naturally occurring methylxanthines with melted DNA using *T_m_/pH* profiles. Furthermore the DNA melting analyses are useful to identify the mutations in cancer samples through high resolution DNA melting profiles methods [32], [33], and useful for the crucial identification of genotyping of human papilloma virus, Lepidopteran and other bacterial models [34]–[36].

Therefore by considering the importance of methylxanthines as modulators of cellular events, the current study enlightens detailed comparative analyses of methylxanthines interaction with DNA with an exploration on their binding activity either in the presence or absence of Mg^2+^ and during helix-coil transitions by *T_m_/pH* melting profiles. Thus understanding the interactions of methylxanthines with DNA as evinced by above methods gain importance mainly because the expression of such nucleic acids functions could easily be modulated by targeting drugs with less cellular toxicities, and that might pave the way for the advantageous innovations of therapeutic interventions.

## Materials and Methods

### DNA and methylxanthines

Lyophilized calf thymus DNA (Sigma, St. Louis, USA) was dissolved in 1× saline sodium citrate (SSC) buffer (0.15 M NaCl and 0.015 M sodium citrate, *pH* 7.5) as 10 mg% solution and left overnight at 37°C with occasional vortexing. Constant concentration of DNA (0.343 O.D./absorbance at 260 nm corresponding to ≈17.2 µg/ml) was maintained for UV absorption studies. On the other hand Herring sperm (HiMedia, Mumbai, India) DNA was used for FTIR (Bruker IFS 66V, Germany) analysis alone. The term drugs used here are with reference to the xanthine derivatives such as theophylline (X1), theobromine (X2) and caffeine (X3) (Sigma, St. Louis, MO, USA).

### UV absorption spectroscopy

For studying interaction of methylxanthines with native form of DNA or *T_m_*-melted DNA, different aliquots of known concentration of DNA (as mentioned above) was taken in DNase/RNase free microcentrifuge tubes, and the drugs were discretely added at different drug-phosphate (P/D) ratios: 0.8, 1.0, 3.0 & 6.0. The final volume was made up to 1 ml using 1× SSC buffer. All the samples were incubated overnight at 37°C. Next day, each sample was repeatedly scanned between 200–300 nm, using Varian, Cary, 1E UV/visible spectrophotometer (Switzerland). However *T_m_*-melted DNA was obtained by heating the mixtures at 100°C and snap cooled. After a brief incubation, scanning was taken between 200–300 nm. The above setup was also studied in the presence of varying concentration of Mg^2+^ (1–10 mM). The spectra of free drugs, free DNA or *T_m_*-melted free DNA were obtained and treated as controls.

### Binding constants

The binding efficacy/activity of these three xanthines with DNA was ascertained at varying drug concentrations in P/D ratios (P/D 0.8, 1.0, 3.0 and 6.0), where the binding constants were obtained as reported [37], [38]. In order to calculate the binding constant (K) for the DNA – methylxanthines (theophylline or theobromine or caffeine) complex, it is alleged that DNA-methylxanthines complex forms in a ratio of 1∶1, based on this the following equations can be established.
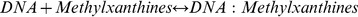
(1)The equilibrium constant is known from
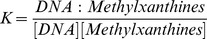
(2)
Equation (2) can be written as
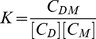
(3)where the C_DM_, C_D_, and C_M_ are the analytical concentrations of DNA-methylxanthines complex, DNA and methylxanthines (theophylline or theobromine or caffeine) respectively.

The Beer Lambert law for the absorption of light is assumed to be followed by the DNA drug binding.
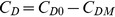
(4)

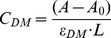
(5)and
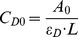
(6)where C_D0_ is the concentration of pure DNA, A_0_ and A are the absorbance (at 260 nm) of pure DNA and in the presence of methylxanthines (theophylline or theobromine or caffeine) respectively. L is the path length of the cuvette i.e. 1 cm.

By incorporating the values of C_D_ and C_DM_ from the above equations into equation (3), the following equation can be derived:
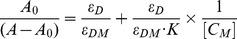
Therefore the double reciprocal plot of 1/(A−A_0_) versus 1/[C_M_] is linear and the binding constant (K) can be estimated by calculating the ratio of the intercept to the slope,
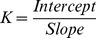
(7)


### Scheming of hyperchromicity

The percentage of hyperchromicity was computed for the binding interaction of xanthine derivatives with DNA at different P/D's using the formula: 100(*A*_260_−*A* ^o^_260_)/(*A*_260max_−*A* ^o^_260_), where *A*_260_ is absorbance at 260 nm at any particular drug concentration, *A* ^o^_260_ is the initial absorbance at 260 nm and *A*_260max_ is the absorbance at maximum drug concentration.

### *T_m_*-melting profile

The absorbance of heat melted DNA in the presence of drugs at varying P/D ratios was compared with heat melted free DNA (without drugs) and double helical (non-heat melted) DNA. The increased binding affinity of the drugs with heat melted DNA was assessed by computing the formula: 100(*NHD*_260_−*A*_260_)/(*T_m_D*_260_−*A*_260_), where *NHD*_260_ is the absorbance (260 nm) of non-heat melted DNA in the presence of particular drug at particular concentration, *T_m_D*_260_ is the absorbance of (260 nm) temperature melted DNA in the presence of particular drug at particular concentration and *A*_260_ is the absorbance of free DNA at 260 nm.

### *pH*-melting profile

As an alternative to *T_m_* study, helix-coil transitions were studied using *pH* as a denaturing parameter. The study was performed with a constant or known concentration of DNA (as mentioned above) taken from the non-buffered stock solution. To produce a *pH*-melting curve, 1 M NaOH solution was added in 1 µl quantity to the cuvette (1 cm optical path) containing the DNA solution in stepwise manner. After adding every µl of NaOH solution, a brief mixing was given and absorbance was subsequently recorded at 260 nm [39]. In *pH* melting study the absorbance reached a plateau by ∼18–20 µl of 1 M NaOH. The *pH* that corresponded to the midpoint between the initial and final absorbance values was taken as the melting *pH*. Accordingly, the melting point was found to be 11.95±0.01. The *pH* melting profile was obtained for a) DNA alone b) DNA with drugs at varying P/D ratios and the percentage of hyperchromicity was computed (at each point of *pH* varying from 1.9 to 19.9) using the formula 100 (*A*_260_−*A* ^o^_260_)/(*A*_260max_−*A* ^o^_260_), where *A*_260_ is absorbance at 260 nm at any particular *pH*, *A* ^o^_260_ is the initial absorbance at 260 nm and *A*_260max_ is the maximum absorbance attained after reaching plateau.

### FTIR spectroscopy

FTIR spectroscopy was employed to study the mode of interaction of theophylline, theobromine and caffeine both in the presence or absence of Mg^2+^ (1–30 mM) with Herring sperm DNA (not highly polymerized). DNA-drug and Mg^2+^-DNA-drugs complexes were prepared and the spectra were obtained with repeated scanning between 1400–400 cm^−1^ according to our published protocol [40].

## Results and Discussion

### Interaction of methylxanthines with native form of DNA: UV absorption

**Figure 2:**
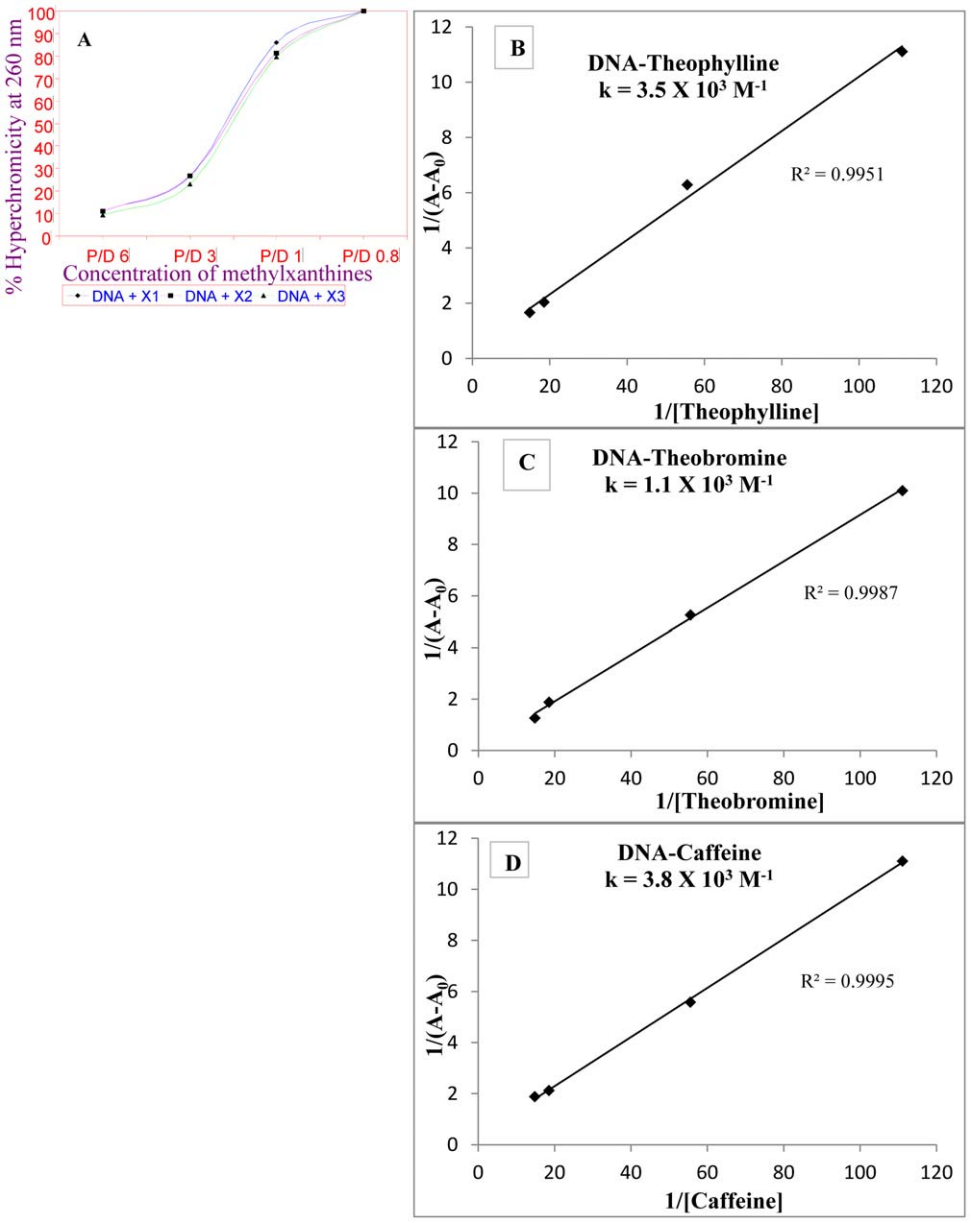
Methylxanthines binding with DNA. (**A**). The percentage of hyperchromicity with respect P/D 6, 3, 1 and 0.8 of theophylline (X1), theobromine (X2) and caffeine (X3) while interacting with DNA. (**B–D**): The double reciprocal plot of 1/(A−A_0_) vs 1/[theophylline or theobromine or caffeine] (**B**) DNA-theophylline complexes; (**C**) DNA-theobromine complexes (**D**) DNA-Caffeine complexes, where A_0_ = absorbance (260 nm) of free DNA, [theophylline or theobromine or caffeine] = concentration of the respective drug.

We examined the changes induced in the UV spectra of calf thymus DNA owing to interaction of xanthine derivatives at different P/D ratios (0.8, 1.0, 3.0 and 6.0). The ultraviolet absorbance for free methylxanthines, free DNA and DNA-methylxanthines complexes were obtained. Based on these spectra, percentages of hyperchromicity (Fig. 2A) and the binding constants of methylxanthines with DNA (Figs. 2B–D) were calculated as described. Figures 2A–D confirm the binding of methylxanthines with DNA, and the binding affinity increased with respect to increasing drug concentration, exhibiting a dose dependent behavioral pattern for DNA binding. The percentage of hyperchromicity indicates a similar fashion or mode of methylxanthines binding with DNA bases. Though the calculated hyperchromicity denotes a similar mode of binding (Fig. 2A), these methylxanthines exhibit a differential binding efficacy with DNA, where caffeine and theophylline show up little higher binding efficacy with DNA than theobromine as predicted by binding constant analysis (Figs. 2B–D). Thus the order of binding affinity is visualized as “**caffeine≥theophylline>theobromine**”.

### Interaction of methylxanthines with native form of DNA: FTIR analysis

**Figure 3:**
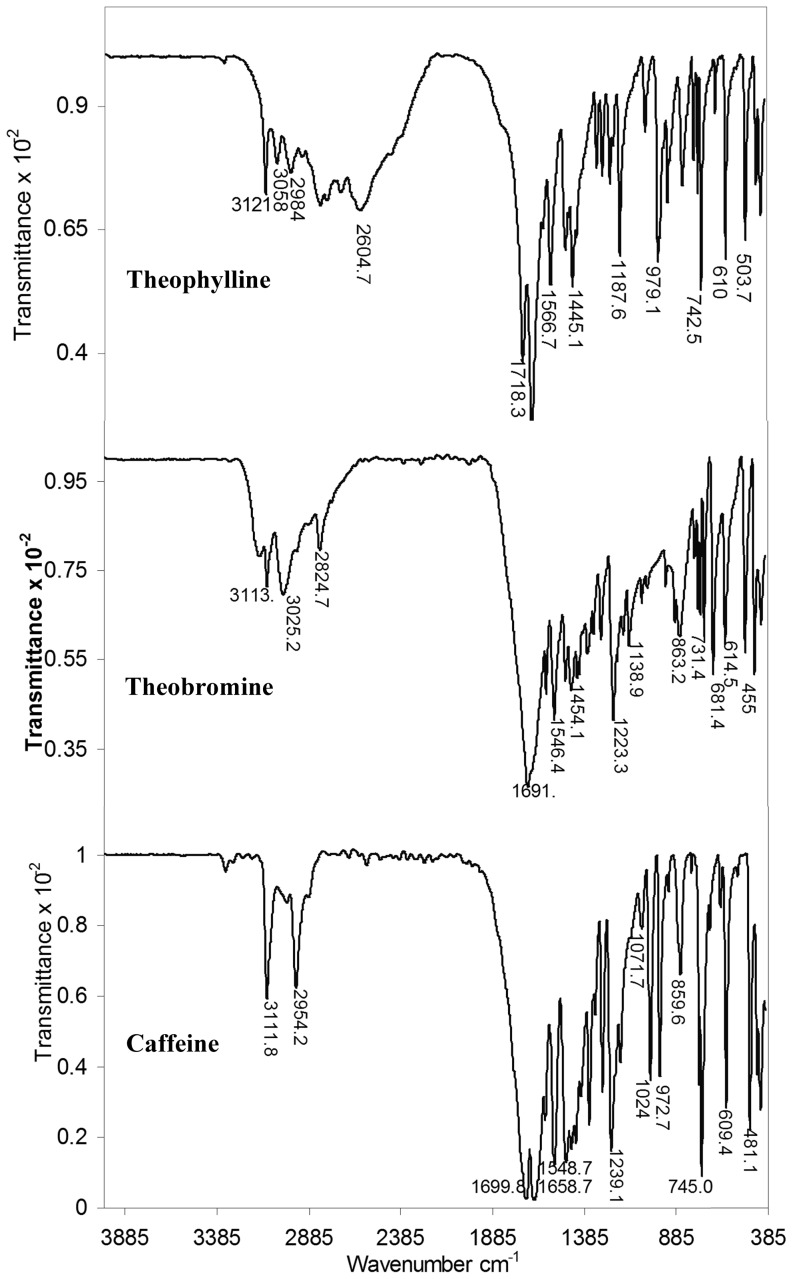
FTIR spectra of free methylxanthines in the region of 1400–400 cm^−1^.

**Figure 4:**
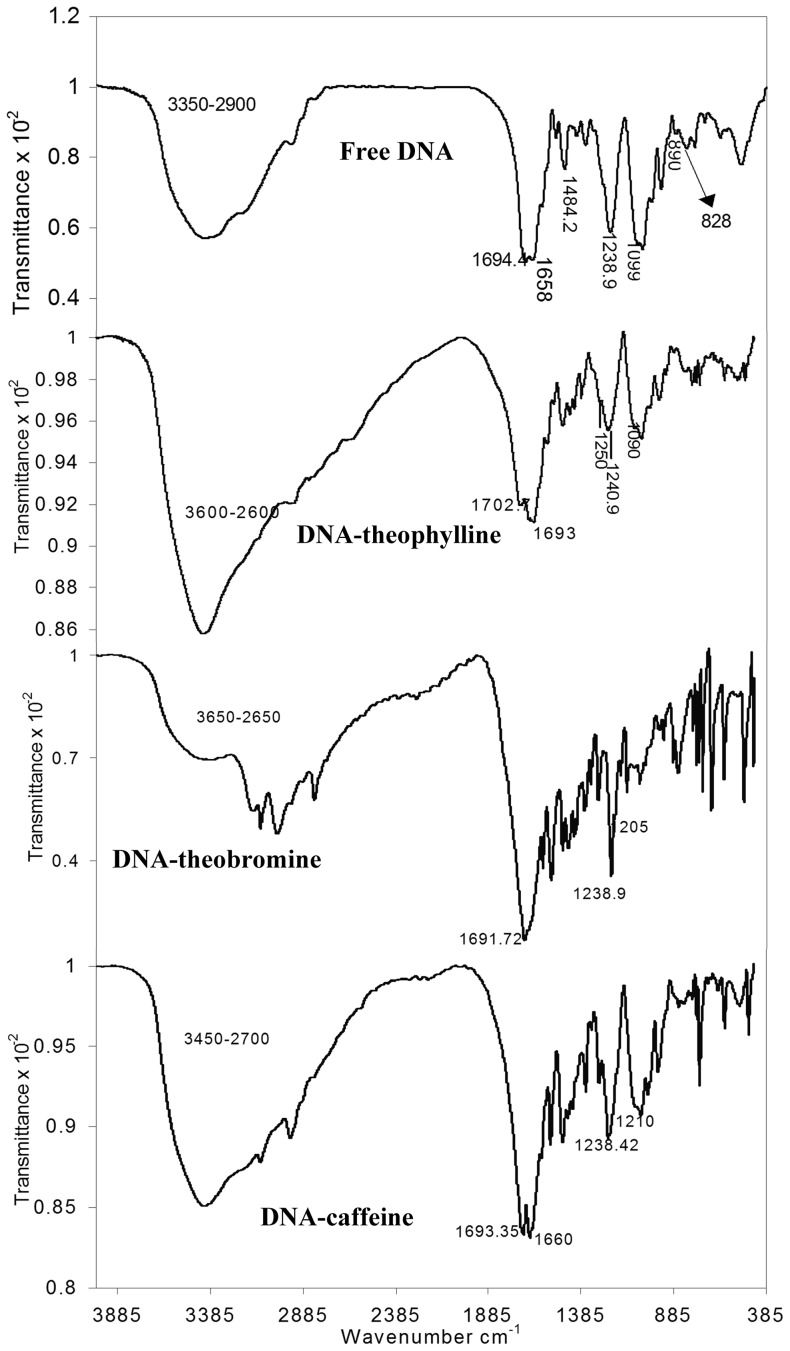
FTIR spectra of free DNA and DNA-methylxanthines complexes obtained with repeated scanning between 1400–400 cm^−1^.

FTIR study was performed to understand the changes in structural features of DNA upon methylxanthines binding. The infrared spectra of DNA-drug complexes offered an evidence for the direct binding of theophylline, theobromine and caffeine to DNA. The FTIR spectra of free drugs are depicted in Fig. 3. The spectra of free DNA and the complexes are shown in Fig. 4. Changes noticed in the functional groups are tabulated for easy reference (Table 1). The vibrational frequency of the imino group (NH) of free DNA appeared around 3550–2900 cm^−1^ (Fig. 4) was changed into 3600–2600, 3650–2650 and 3450–2700 cm^−1^ in DNA-theophylline, DNA-theobromine and DNA-caffeine complexes respectively (Fig. 4) (Table 1). This obviously indicates that the band responsible for the imino group of free DNA became broadened upon drug complexation. The broadening of NH band in complexes is mainly attributed to the interaction of drugs with DNA bases through H-bonding.

**Table 1:** The vibrational frequencies of C = O, NH and PO^−^_2_ (FTIR, KBr cm^−1^) bands of free DNA, free drugs and DNA-drug complexes.

Functional Groups	Free DNA (cm^−1^)	Free Drugs (cm^−1^)			DNA-X1 (cm^−1^)	DNA-X2 (cm^−1^)	DNA-X3 (cm^−1^)
		X1	X2	X3			
NH	3350–2900	3121	3113	3111	3600–2600	3650–2650	3450–2700
C = O	1694.4	1718, 1666.8	1691.7	1699.8, 1658.7	1702.7	1691.7	1693.35
PO^−^_2_ (υ_as_)	1238.9	—	—	—	1250, 1240.9	1223.83, 1205	1238.42, 1210
PO^−^_2_ (υ_s_)	1099	—	—	—	1090	1070.95	1098

X1 = theophylline, X2 = theobromine and X3 = caffeine.

DNA-X1 = DNA-theophylline complex, DNA-X2 = DNA-theobromine complex and DNA-X3 = DNA-caffeine complex.

It is observed that the carbonyl (C = O) vibration frequency (υ_C = O_) of both drug (theophylline = 1718, 1666 cm^−1^; caffeine = 1699.8, 1658.7 cm^−1^) as well as DNA (1694.4 cm^−1^) disappeared and a new vibration band with change in the intensity appeared around 1702.7 and 1693.35 cm^−1^, in DNA-theophylline and DNA-caffeine complexes respectively. This indicates that the C = O and NH groups of drug and DNA are effectively involved in H-bonding interaction. However, we noticed only minor change in the C = O frequency of theobromine (1691.7 cm^−1^) (Table 1).

The H-bonding interaction of methylxanthines with DNA bases (G-C/A-T) is substantiated by the major spectral changes of DNA in-plane vibrations in the region of 1707–1400 cm^−1^ [41], [42]. The band at 1694.4 cm^−1^ (G, T) related to mainly guanine shifted to 1702.7, 1691.72 and 1693 cm^−1^ in DNA-theophylline, DNA-theobromine and DNA-caffeine complexes respectively. The changes were also observed in the band at 1658 cm^−1^ (T, G, C) mainly for thymine [41], [42], cytosine band at 1484.2 cm^−1^ (C, G) and for adenine at 1600 cm^−1^ upon drug complexation. The shifting in the vibrational frequency of bases due to drug complexation clearly indicates that methylxanthines are able to interact with A-T and G-C bases where the NH and C = O of DNA and drugs are mutually involved in H-bonding. This gains the support from the studies of Nafisi et.al, that caffeine and theophylline complexations with DNA are established through the hydrogen bonding interaction [17].

Also splitting/shifting was observed in the vibrational intensities of assymetric (υ_as_) and symmetric (υ_s_) PO^−^_2_ band of free DNA at 1238.9, 1099 cm^−1^ respectively. In DNA-theophylline complexes the PO^−^_2_ band of free DNA at 1238.9 cm^−1^ exhibited splitting and shifting into 1250 and 1240.9 cm^−1^. Similarly in DNA-theobromine and DNA-caffeine complexes the PO^−^_2_ band exhibited shifting into 1223.83, 1205 cm^−1^ and 1238.42, 1210 cm^−1^ respectively. Whereas the υ_s_PO^−^_2_ band of free DNA at 1099 showed shifting into 1090, 1070.95 and 1098 in DNA-theophylline, DNA-theobromine and DNA-caffeine complexes respectively (Table 1). The changes observed in the vibrational frequency of PO^−^_2_ band are attributed to the possible interaction of methylxanthines with DNA bases as well as to the phosphate groups. At the same time only minor perturbations were observed in the IR marker bands of free DNA, sugar-phosphate stretch (890 cm^−1^) and phosphodiester mode (828 cm^−1^) upon drug interaction and hence the DNA remained in the B-family structure in complexes or very partial structural changes were noticed. Hence based on the FTIR analyses the order of DNA binding affinity is visualized as “**caffeine≥theophylline>theobromine**”, and it is correspondingly similar with that of the binding constants derived from UV analysis.

On the other hand Nafisi et.al. have shown that theophylline binds to DNA with more efficacy than the caffeine [17]. The current study indicates more or less an equal efficacy for theophylline and caffeine than theobromine. Minor variations are observed for UV and FTIR analyses with that of Nafisi et.al for caffeine and theophylline complexations with DNA. This is mainly due to different concentrations of caffeine and theophylline used for DNA complexation, and especially the FTIR that we have performed are of solid-state analysis [40]. Generally IR spectra for DNA and its ligand complexes are taken in solution [17] but in our case IR spectra for DNA-drug complexes are studied in solid-state using KBr pellet as reported from our previous works [40], and also the DNA used here for the FTIR study was not highly polymerized and hence the minor variation in the IR transmittance for PO^−^_2_ stretch and other marker bands by 10–20 cm^−1^.

### Interaction of methylxanthines in the presence of Mg^2+^ with DNA: UV absorption

**Figure 5:**
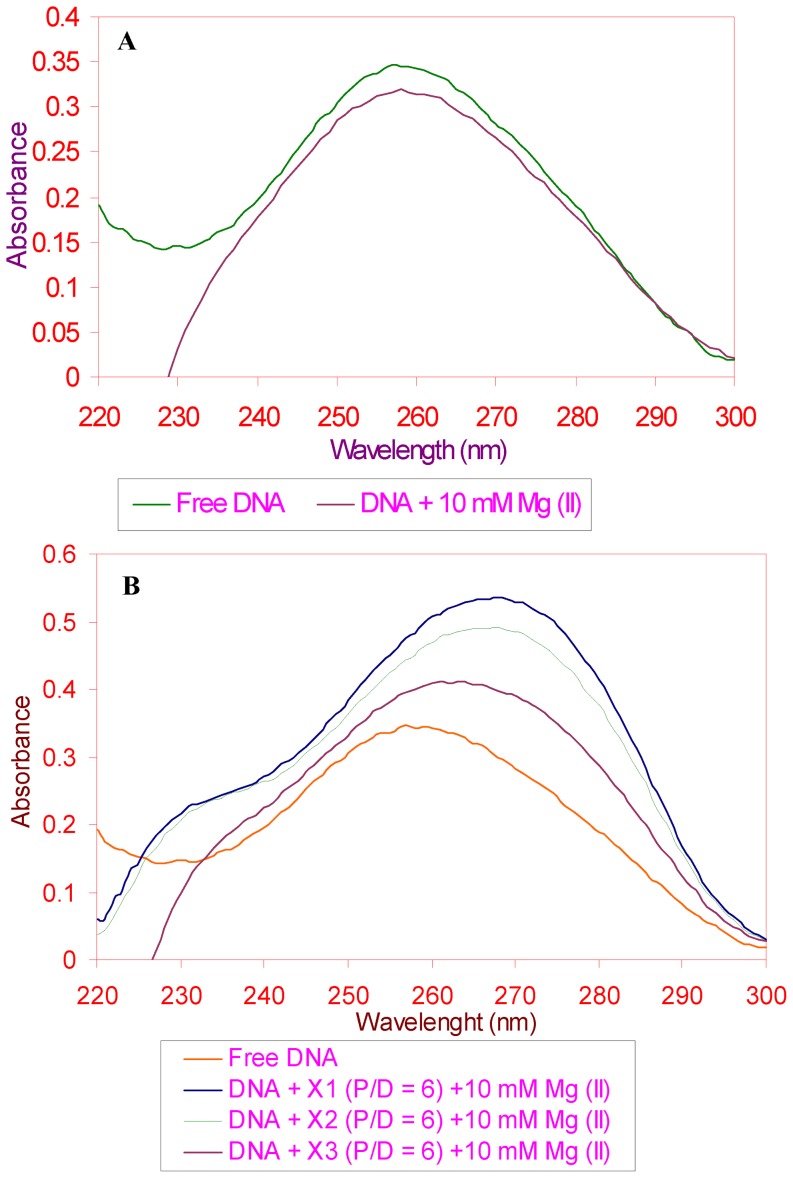
Binding affinity of methylxanthines in the presence of divalent metal ion. (**A**). Ultraviolet absorption spectrum of DNA in the presence of 10 mM Mg^2+^. (**B**). Changes in the methylxanthines (theophylline, theobromine and caffeine) bound (P/D 6) DNA spectra in the presence of 10 mM Mg^2+^.

Changes in the UV spectra of calf thymus DNA induced by methylxanthines were monitored in the presence of Mg^2+^. This was carried out, both, as a function of magnesium and methylxanthines concentration. The interaction was studied in different P/D's and MgCl_2_ concentrations, however we here display the changes observed at 10 mM MgCl_2_ and at P/D's 6 as representative for this particular study. Initially we observed a hypochromic shift in DNA-Mg^2+^ mixtures (without drugs) (Fig. 5) but intriguingly this was reverted to hyperchromicity at various P/D's concentration and the one that is depicted here is P/D 6 in the vicinity of 10 mM MgCl_2_ (Figs. 5A–B).

Before studying the UV spectra of calf thymus DNA with different concentration of xanthine derivatives either in the presence or absence of divalent metal ions, the native spectra of all the three drugs used for the binding interactions were also studied. The absorption maxima for theophylline, theobromine and caffeine were found to lie in the region of 269–278 nm (λ_max_: ∼274 nm) (figures not included). During the binding interaction of these xanthines with DNA either in the presence (Fig. 5) or absence of divalent metal ions such as Mg^2+^, DNA spectra exhibited a shift in nm, where the free DNA absorbance λ_max_ at 260 nm, shifted to 270 nm in DNA-drug or DNA-drug-metal complexes with a prominent hyperchromicity. The shift in the nm signifies the formation of binding adducts for DNA-drug complexes (with or without metal) at 270 nm (λ_max_). This 270 nm somewhat closer to the λ_max_ of native drug spectra, indicating all these methylxanthines interact with DNA bases from outside to the DNA double helix with certain level of masking the DNA. In other words this is referred as ‘*DNA masking effect*’ by methylxanthines, which are noticed at higher concentration of drug interaction. Other mode of interaction with DNA structure such as the intercalation (inside the helix) could not be a predominant interaction for methylxanthines binding with DNA [2]. Though UV absorption did point to the role of backbone mediated interaction of metal with DNA as well as the interaction of drugs with DNA in the vicinity of metal, more detailed analysis rendered by FTIR spectroscopy reveals the differential binding of methylxanthines as detailed below.

### Interaction of methylxanthines in the presence of Mg^2+^ with DNA: FTIR analysis

**Figure 6:**
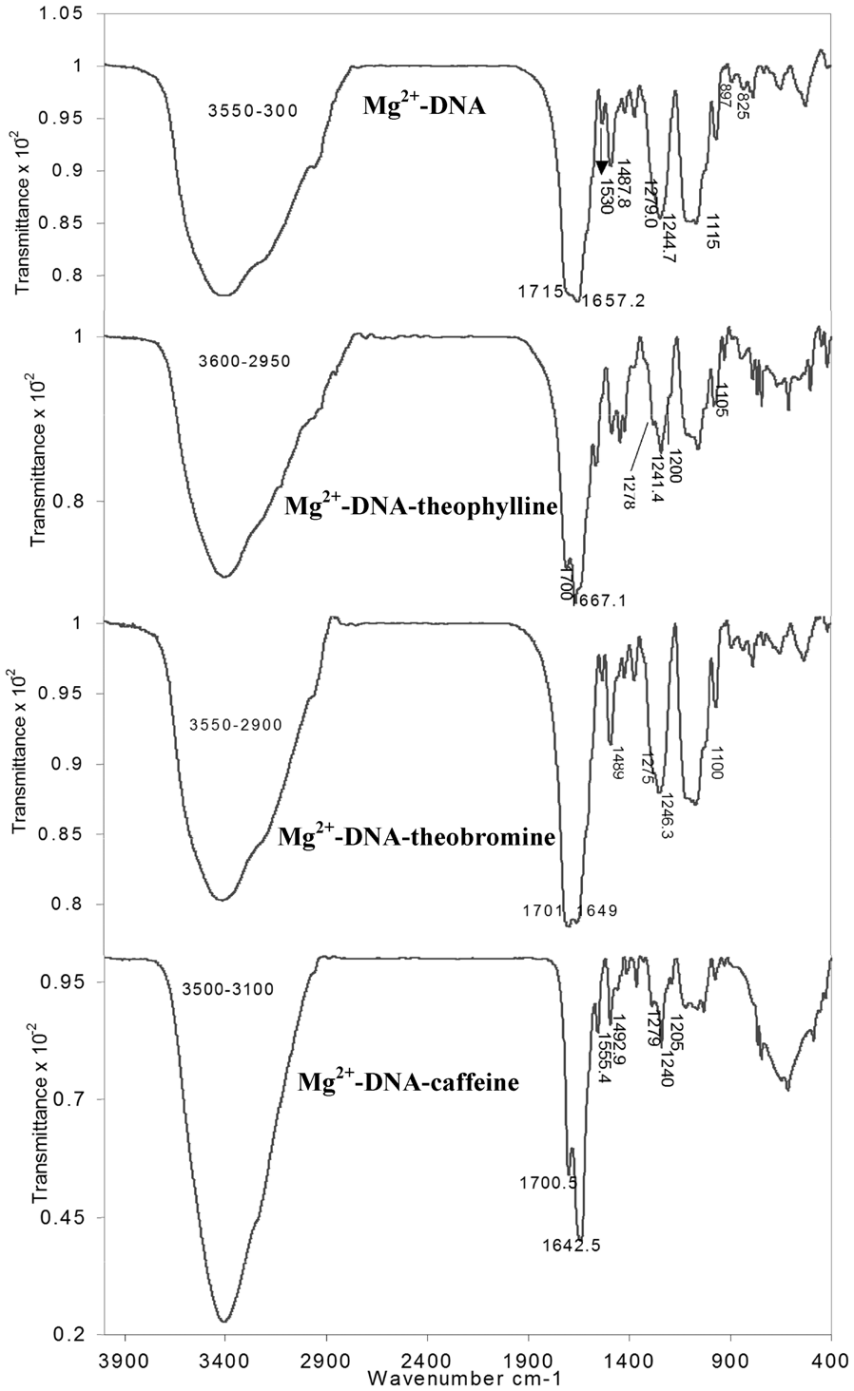
FTIR spectra of DNA, DNA-methylxanthines complexes in the presence of Mg^2+^ (30 mM).

The main IR spectral features related to DNA-Mg^2+^, DNA-Mg^2+^-drug complexes are shown in Fig. 6. If required, these Figures can also be compared with the free DNA, free drugs and non-metal DNA-drug complexes (Figs. 3 and 4). Also for a quick reference the changes in the functional groups are tabulated (Table 2). We examined the spectral changes of DNA and drugs in the presence of Mg^2+^ from 1–30 mM concentration. However, significant changes were observed in the FTIR spectra of drug complexed DNA in the presence of Mg^2+^ at 30 mM concentration and the details are discussed below.

**Table 2:** The vibrational frequencies of C = O, NH and PO^−^_2_ (FTIR, KBr cm^−1^) bands of free DNA, free drugs and DNA-drug-metal complexes.

Functional Groups	Free DNA (cm^−1^)	Free Drugs (cm^−1^)			Mg(II)- DNA (cm^−1^)	Mg(II)- DNA-X1 (cm^−1^)	Mg(II)- DNA-X2 (cm^−1^)	Mg(II)- DNA-X3 (cm^−1^)
		X1	X2	X3				
NH	3350–2900	3121	3113	3111	3550–3000	3600–2950	3550–2900	3500–3100
C = O	1694.4	1718, 1666.8	1691.7	1699.8, 1658.7	1715	1700	1701	1700.5
PO^−^_2_ (υ_as_)	1238.9	—	—	—	1279, 1244	1278, 1241.4, 1200	1275, 1246.3	12791, 1240, 1205
PO^−^_2_ (υ_s_)	1099	—	—	—	1115	1105	1100	1120

X1 = theophylline, X2 = theobromine and X3 = caffeine.

Mg(II)-DNA = Mg^2+^-DNA complex, Mg(II)-DNA-X1 = Mg^2+^-DNA-theophylline complex, Mg(II)-DNA-X2 = Mg^2+^-DNA-theobromine complex and Mg(II)-DNA-X3 = Mg^2+^-DNA-caffeine complex.

The υ_as_/υ_s_ PO^−^_2_ band of free DNA at 1238.9 and 1099 cm^−1^ showed variation due to Mg^2+^ interaction. In DNA-Mg^2+^ complex, the band at 1238.9 cm^−1^ exhibited shifting and splitting into higher frequency at 1279 and 1244 cm^−1^, whereas in Mg^2+^-DNA-theophylline and Mg^2+^-DNA-caffeine complexes the band showed shifting and splitting into three components at 1278, 1241.4, 1200 cm^−1^ and 1279, 1240, 1205 cm^−1^ respectively. For Mg^2+^-DNA-theobromine complexes the band showed splitting at 1275 and 1246.3 cm^−1^. Also changes in the υ_s_ PO^−^_2_ band of the free DNA at 1099 were noticed in DNA-Mg^2+^ (1115 cm^−1^), Mg^2+^-DNA-theophylline (1105 cm^−1^), Mg^2+^-DNA-theobromine (1100 cm^−1^) and Mg^2+^-DNA-caffeine (1120 cm^−1^) complexes (Table 2) (Fig. 6). The PO^−^_2_ band was observed at higher frequency in DNA-Mg^2+^ complexes, indicating strong metal coordination to DNA phosphates. The shifting observed in the PO^−^_2_ band of DNA-Mg^2+^ complexes was little high when compared to the free DNA. This is because of the fact that the complexation of Mg^2+^ was obtained in solid state avoiding H_2_O completely. This shifting may not be observed in solution spectra, where the Mg^2+^ coordination always be mediated through water molecules leading to the reduced impact on DNA phosphates, whereas in solid state, coordination of metal leads to higher impact and hence the discrepancy in PO^−^_2_ band shifting.

It was observed that the band at 1694.4 cm^−1^ (υ_C = O_) for free DNA exhibited shifting at 1715 cm^−1^ in DNA-Mg^2+^ complexes. The shifting in the vibrational stretching frequency of C = O in DNA-Mg^2+^ complexes is mainly attributed to the metal coordination with N7 guanine, N3 cytosine, thymine O2 and adenine N7. A similar kind of observation substantiates the above interaction [41], [42]. Interestingly, in the presence of Mg^2+^, the C = O vibrational frequency of both drug and DNA disappeared and shifted to higher frequency at 1700, 1701, 1700.5 cm^−1^ in Mg^2+^-DNA-theophylline, Mg^2+^-DNA-theobromine and Mg^2+^-DNA-caffeine complexes correspondingly (Table 2) (Fig. 6), indicating the enhanced binding of these drugs in the presence of Mg^2+^.

The broadening of NH peak as observed as function of intramolecular H-bonding in free DNA (3600–2900 cm^−1^) (Fig. 4) was reduced in DNA-Mg^2+^ complexes (3550–3000 cm^−1^) (Fig. 6) (Table 2). The intramolecular H-bonding reduction by Mg^2+^ can be attributed to its coordination with DNA phosphates and also to N7 adenine/guanine, thymine O2 and N3 cytosine. The coordination effected by Mg^2+^ could be seen by comparing the vibrational stretching frequencies of C = O and PO^−^_2_ bands in DNA-Mg^2+^ complexes. Intriguingly, the broadening effect was restored or reverted back to certain extant in Mg^2+^-DNA-theophylline (3600–2950 cm^−1^), Mg^2+^-DNA-theobromine (3550–2900 cm^−1^) and Mg^2+^-DNA-caffeine (3500–3100 cm^−1^) complexes (Fig. 6) (Table 2), signifying that the reduced intramolecular H-bonding by Mg^2+^ favors the enhanced binding of methylxanthines with DNA through H-bonding interaction. In addition to the NH band, support for the enhanced binding of methylxanthines with DNA also comes from a) the changes in C = O vibrational frequency observed at 1715 cm^−1^ of DNA-Mg^2+^ complexes b) shift in the bands of DNA bases (described below).

The enhanced binding of methylxanthines with DNA in the vicinity of Mg^2+^ gains support due to shift in the bands of DNA bases or DNA in-plane vibrations in the region of 1707–1400 cm^−1^ [41], [42]. The band at 1707.3 cm^−1^ (G, T) related to mainly guanine shifted to 1715, 1700, 1701 and 1700.5 in Mg^2+^-DNA, Mg^2+^-DNA-theophylline, Mg^2+^-DNA-theobromine and Mg^2+^-DNA-caffeine complexes respectively. The changes observed in the band at 1658 cm^−1^ (T, G, C) mainly for thymine [41], [42], cytosine band at 1484.2 cm^−1^ (C, G) and for adenine at 1600 cm^−1^ upon drug complexation, indicating binding of methylxanthines were greatly enhanced in the presence of Mg^2+^. Especially theobromine binding was improved when compared to its non-metal complexes, where a minor change alone was noticed in the C = O frequency of drug (Fig. 3 and 4).

Together with the changes observed in the PO^−^_2_ band of DNA during complexation with metal and drugs, changes were also observed in the main IR marker bands at 890 cm^−1^ (sugar-phosphate stretch) and 836 (phosphodiester mode). These IR marker bands showed some variations in complexes at 897, 825 cm^−1^ (Mg^2+^-DNA); 898 cm^−1^ (Mg^2+^-DNA-theophylline); 895, 830 cm^−1^ (Mg^2+^-DNA-theobromine) and 898, 832 cm^−1^ (Mg^2+^-DNA-caffeine). Hence the DNA structure was shifted from B family to A- family in the above complexes. Other than the structural alteration, the changes in the PO^−^_2_ band of DNA can also be attributed to the metal interaction with N7 adenine/guanine, thymine O2 and N3 cytosine. Here the study encompassing the drug interaction in the presence of lower metal ion concentration (1, 5 and 10 mM) did not show any major shifting as explained above and resembled as that of DNA-drug complexes in the absence of metal ions.

### Interaction of methylxanthines during helix-coil transitions of DNA by *T_m_/pH* melting profiles

**Figure 7:**
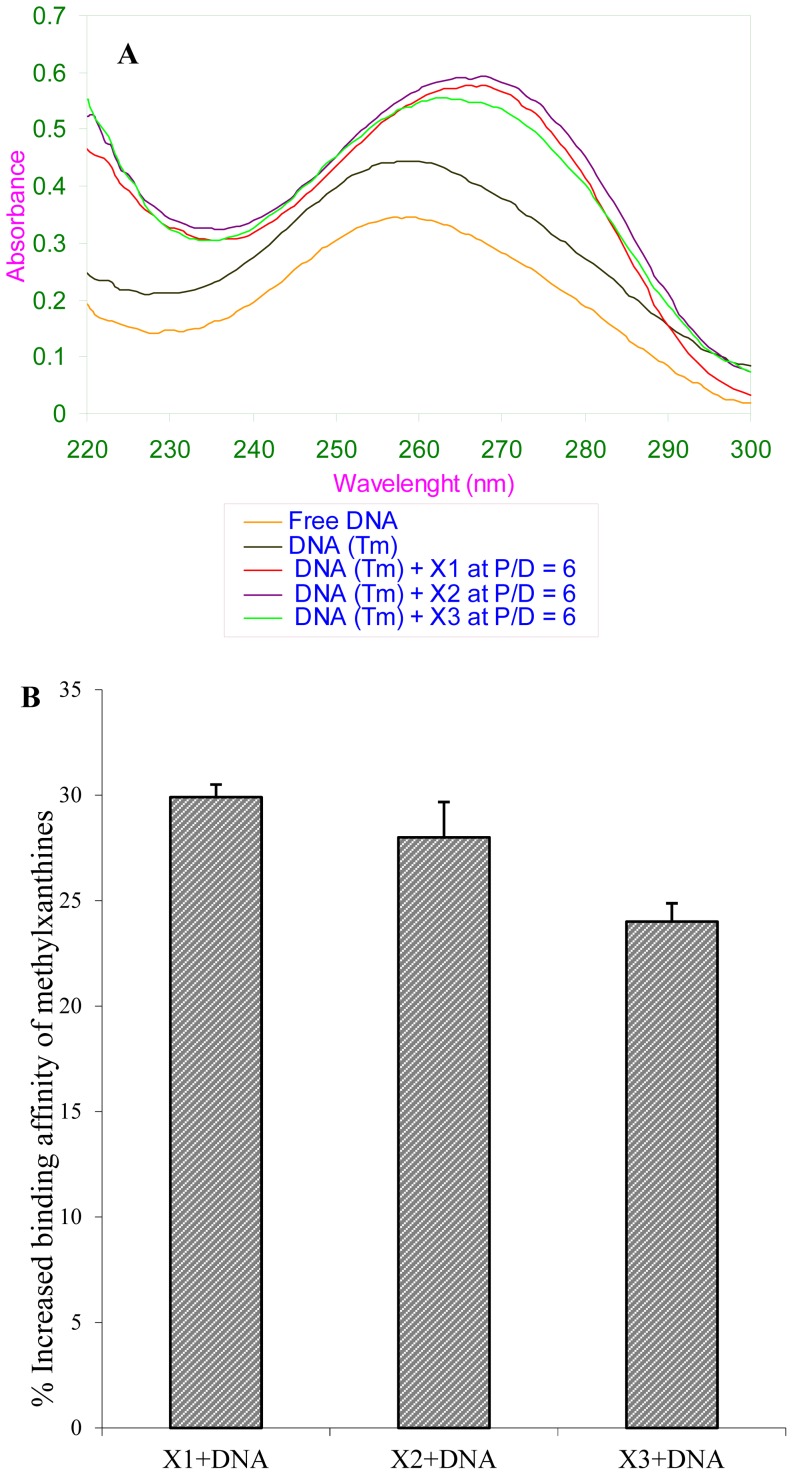
Helix-coil transition analysis, *T_m_-*profile. (**A**). UV spectra of *T_m_*-DNA in the presence of methylxanthines at P/D 6. (**B**). A representative picture indicating the percentage of increased binding activity of methylxanthines with *T_m_-*DNA at P/D 6. X1-theophylline, X2-theobromine and X3-caffeine. Values are mean ± SE with p<0.002 vs control.

Interaction of methylxanthines was studied during helix-coil transition of DNA, using higher temperature and *pH a*s described in the methods section. The absorbance spectrum of heat melted DNA in the presence of drugs at varying P/D ratios was compared with heat melted free DNA (without drugs) and double helical (non-heat melted) DNA. *T_m_* melting profile showed a significant increase of 24–30% in binding activity of methylxanthines (P/D 6) with DNA during helix-coil transition state rather than to a native double helical structure or in absence of any helix-coil transitions (Figs. 7A–B).

**Figure 8:**
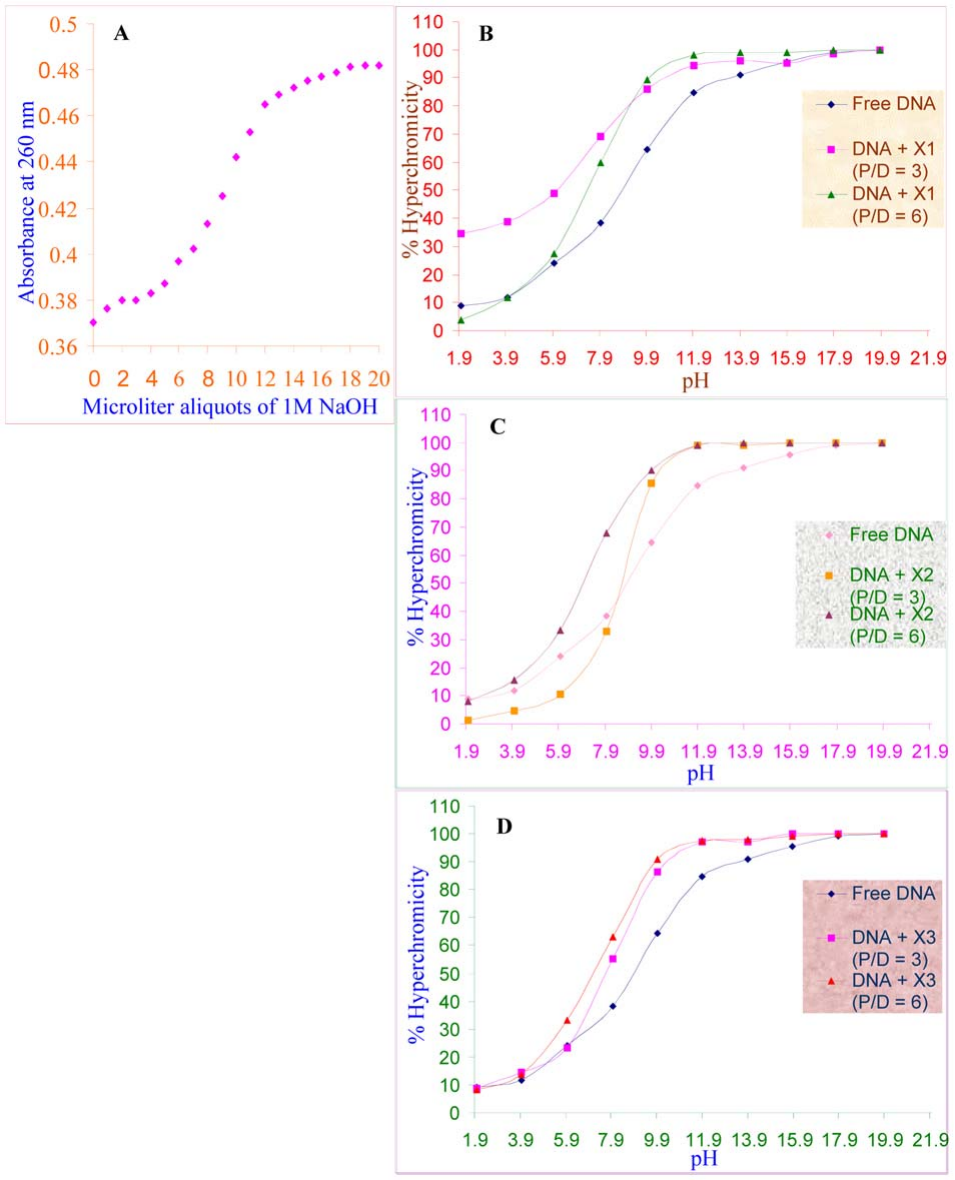
Helix-coil transition analysis, *pH* melting profile. (**A**). *pH* melting profile of calf thymus DNA in 10 mM NaCl, 25 µM EDTA, produced by adding µl aliquots of 1M NaOH. *pH* melting curves of calf thymus DNA, which was preincubated with theophylline (X1) (**B**), theobromine (X2) (**C**) and Caffeine (X3) (**D**) at P/D 3 and 6.

Furthermore, the helix-coil transition observed using *pH* variation method in the presence and absence of drugs as well revealed an increased binding affinity of methylxanthines with DNA. This is supported by the fact that the percentage of hyperchromicity of the free DNA was increased with respect to its state of helix-coil transition (due to slow increase in *pH*), and intriguingly the percentage of hyperchromicity of free DNA was still more increased to 30–35% upon addition of methylxanthines (P/D: 3 and 6), supporting the enhanced binding activity of methylxanthines during helix-coil transition of DNA (Figs. 8A–D). The above findings (*T_m_/pH* melting profiles) suggest the preferential binding of methylxanthines to single stranded DNA rather than to a native double helical.

### Binding affinity of methylxanthines

Interestingly there is a binding affinity difference with DNA is noticed for these three methylxanthines in the presence of divalent metal ions and with heat or *pH* melted DNA as compared to that of the native double helical DNA. A prominent increase in the binding efficacy is noticed for theophylline and theobromine than caffeine (Figs. 5, 7 and 8) in the above set up. This suggests that caffeine interacts with double helical DNA (Fig. 2D) by establishing H-bonding interaction from outside to DNA helix and forming aggregation along the sides of DNA polymer [2], [3]. However caffeine interaction with the denatured form of DNA (*T_m_/pH*-melted) (DNA structure closer to single strand) or in the presence of divalent metal ions reveals lesser binding activity for caffeine (Figs. 5, 7 and 8). This could be substantiated by the fact that the binding affinity of these xanthine derivatives were enhanced with respect to the degree of exposure of DNA bases to give rise more binding sites for drugs. This in turn renders the binding efficacy to increase for each methylxanthines; and at the same time the presence of bulky methyl groups (1,3,7-trimethyl) in caffeine (Fig. 1) impede its efficacy and thereby theophylline or theobromine exceeds caffeine in the binding affinity. Thus the steric hindrance offered by methyl groups in methylxanthines are considered to be the rate limiting factors in determining its preferential or increased binding affinity with *T_m_* or *pH* melted DNA. This observation is very much similar to our earlier reported study of RNA binding efficacy of methylxanthines [15], where theophylline and theobromine are shown to have enhanced binding efficacy than caffeine. This binding affinity difference led these molecules to interfere differently in modulating the splicing mechanism of group I intron RNA [25]. As far as the metal ion is considered it reduces the aggregation and induce some structural perturbations in DNA (refer the FTIR analysis above) favor the enhanced binding of methylxanthines that eventually upheave the binding affinity of theophylline and theobromine than caffeine. Hence the order of binding affinity of these methylxanthines with denatured the form of DNA and in the presence of metal ions is visualized as “**theophylline≥theobromine>caffeine**”.

Moreover it is needed to be clarified that even though the *T_m_ or pH* melting directs the native double helical DNA to undergo helix-coil transitions, at some extant re-annealing of DNA is always be un-avoided. However the present study indicates that some degree of re-annealing occurrence together with helix-coil transitions has not affected the overall binding activity of the methylxanthines. Indeed it helped us to study the binding activity of methylxanthines from double helical form of DNA to its denaturing state and enabled to derive and compare the increased binding activity of methylxanthines without changing the transition environment. This is mainly because of the fact that the binding activity of methylxanthines originally refereed to the double helical DNA would be invalid and may not that ease or reliable to compare and derive its increased binding activity in the case of pure form of single stranded DNA environment.

Thus the understanding of nucleic acid structure and their interactions with small molecule drugs as evinced by above methods gain importance mainly because of targeting drugs of our interest could easily modulate the expression of nucleic acids functions. As these naturally occurring methylxanthines are the derivatives of xanthines and/or base analogs of purine nucleotides, the present study accentuated for its interaction with DNA both in the presence and absence of divalent metal ions or during helix-coil transitions depicting a platform for the development of methylxanthines as co-enhancers for targeted drug delivery and therapeutic innovations.
